# Transcriptome analysis of differential sugar accumulation in the developing embryo of contrasting two *Castanea mollissima* cultivars

**DOI:** 10.3389/fpls.2023.1206585

**Published:** 2023-06-19

**Authors:** Ruimin Huang, Fei Peng, Dongsheng Wang, Fei Cao, Chunlei Guo, Liyang Yu, Jingzheng Zhang, Yuedong Yang

**Affiliations:** ^1^ Engineering Research Center of Chestnut Industry Technology, Ministry of Education, Hebei Normal University of Science and Technology, Qinhuangdao, Hebei, China; ^2^ Hebei Key Laboratory of Active Components and Functions in Natural Products, Hebei Normal University of Science and Technology, Qinhuangdao, Hebei, China; ^3^ Hebei Collaborative Innovation Center of Chestnut Industry, Qinhuangdao, Hebei, China; ^4^ Hebei Key Laboratory of Horticultural Germplasm Excavation and Innovative Utilization, College of Horticulture Science and Technology, Hebei Normal University of Science and Technology, Changli, Hebei, China

**Keywords:** embryogenesis, sucrose, starch metabolism, cold, ABA

## Abstract

Chinese chestnut (*Castanea mollissima*) is an important nut tree species, and its embryo is rich in sugar. We combined metabolomic and transcriptomic data to analyze metabolites and genes related to sugar in two Chinese chestnut cultivars at 60, 70, 80, 90 and 100 days after flowering (DAF). The soluble sugar content of high-sugar cultivar at maturity is 1.5 times that of low-sugar cultivar. Thirty sugar metabolites were identified in embryo, with the most dominant being sucrose. Analysis of the gene expression patterns revealed that the high-sugar cultivar promoted the conversion of starch to sucrose by up-regulating genes related to starch degradation and sucrose synthesis at 90-100 DAF. It also strongly increased the enzyme activity of SUS-synthetic, which may promote sucrose synthesis. Gene co-expression network analysis showed that ABA and peroxide were related to starch decomposition during Chinese chestnut ripening. Our study analyzed the composition and molecular synthesis mechanism of sugar in Chinese chestnut embryos, and provided a new insight into the regulation pattern of high sugar accumulation in Chinese chestnut nuts.

## Introduction

Chestnut belongs to the Fagaceae family, which is mainly cultivated in China, Bolivia, Turkey, the Republic of Korea and Italy (Massantini et al., 2021). Chestnuts are rich in starch, minerals, vitamins and phytonutrients, relatively low in fat, and gluten free, making them a healthy and nutritious food (Warmund, 2011; Haytowitz et al., 2018). The composition of carbohydrates has an important effect on the quality of chestnuts. More than 70 percent of respondents said that taste was the most important factor in their decision to buy chestnuts, and sensory analysis showed that chestnut acceptance was strongly dependent on sweetness and related to sucrose content (Künsch et al., 2001; Santos et al., 2022).

Starch is the main form of carbohydrates in chestnuts, and it can be transformed into each other with sucrose (Huang et al., 2021). Sugar and starch metabolism depends on the correct spatial and temporal activity of many enzymes (Chen et al., 2021; Samkumar et al., 2022). During nut development, sucrose is transported to the embryos as a product of photosynthesis. Sucrose can also be converted to glucose and fructose by cytosolic invertase (CINV), or to fructose and uridine diphosphate glucose (UDPG) by sucrose synthase (SUS) (Wan et al., 2018; Moshchenskaya et al., 2019). Then, ADP-glucose (ADPG) is formed through a multi-part reaction and is the common precursor for the synthesis of amylose and amylopectin (Streb and Zeeman, 2012). The synthesis of amylose is catalyzed by granule-bound starch synthase (GBSS). In addition, the synthesis of amylopectin is catalyzed by soluble starch synthetase (SS), starch branching enzyme (SBE) and Isoamylase type starch debranching enzyme (ISA) (Yang et al., 2022).

On the other hand, starch stored in plants will be decomposed into soluble sugar for life activities. Glucan water dikinase (GWD) and phosphoglucan water dikinase (PWD) can phosphate starch and loosen the structure of starch granules in plastid (Mahlow et al., 2016; Xu et al., 2017). Phosphorylated starch can be degraded by a set of glucan-hydrolyzing enzymes (i.e., AMY and BAM) to produce maltose and glucose. Both maltose and glucose can be transported into cytosol to synthesize UDPG (Zhang et al., 2014; Schreier et al., 2019). Some important enzymes such as sucrose phosphate synthase (SPS), sucrose phosphate phosphatase (SPP) and SUS can catalyze UDPG to generate sucrose (Maloney et al., 2015).

Sugar metabolism is regulated by many factors and is easily affected by environment. Low temperatures can destroy the balance of starch and sucrose metabolism, and promote the starch degradation into soluble sugar (Zhang et al., 2014). The contents of glucose, fructose, sucrose, raffinose and galactitol in *Arabidopsis* leaves increased after cold acclimation (Bieniawska, 2007). ABA may be one of the important hormones regulating fruit ripening, which may accelerate sucrose accumulation and promote fruit ripening quality by increasing the enzyme activity of SUS (Wang et al., 2017). Exogenous ABA can increase soluble sugar content in apple fruits at mature stage, and the appropriate concentration of ABA can accelerate glucose conversion and promote sucrose accumulation, which is consistent with the trend of sugar conversion at mature stage (Xiao et al., 2018).

Chinese chestnut is a traditional nut of China and is widely cultivated in East Asia (LaBonte et al., 2018). Hebei Province is the main producing area of Chinese chestnut in China. The four seasons of Hebei Province are clear, and the unique climate may affect the quality of Chinese chestnut (He et al., 2021). In this study, we studied the changes in gene expression related to sugar in the embryos of two Chinese chestnut cultivars, and compared the composition of sugar in the five developmental stages. As a result, the mechanism of high sugar accumulation of Chinese chestnuts was analyzed, and it provided prospects for improving the sugar content of Chinese chestnut cultivars.

## Materials and methods

### Plant material

Two Chinese chestnut cultivars, high-sugar cultivar ‘Yanlong’ (HS) and relatively low-sugar cultivar ‘Yanshanzaofeng’ (LS), were used as plant materials. The nuts of two Chinese chestnut cultivars were collected from the six 11-year-old trees at the Chinese chestnut base in Hebei, China (118°80′E, 40°13′N). Each tree received standard agronomic practices. ‘Yanshanzaofeng’ (LS) has female flowers in full bloom on June 5, and ‘Yanlong’ (HS) has female flowers in full bloom on June 25. Flowers that were at the anthesis stage simultaneously were marked, and fruits were collected at 60, 70, 80, 90, and 100 DAF (fruit maturity) in 2021. At each stage of development, nine nuts were mixed into a biological repetition from the selected three trees, with three repetitions per each stage, and then samples were quickly frozen with liquid nitrogen and stored in a refrigerator at -80°C.

### Analysis of sugar content

The freeze-dried samples were ground into powder with a mixer mill (MM 400, Retsch) by filtration through a 0.5 mm filter. The sucrose content (about 50 mg of sample powder) was determined using the anthrone colorimetric method (Roggo et al., 2002). The contents of starch components (amylose and amylopectin) were determined through dual-wavelength spectroscopy, with amylopectin measured at 550 nm and 695 nm and amylose measured at 617 nm and 475 nm, using a spectrophotometer (UV-2102C) (Juliano et al., 1981). All the determinations were performed in triplicate.

### GC-MS analysis of sugar metabolites

Agilent 8890 gas chromatograph coupled to a 5977B mass spectrometer with a DB-5MS column (30 m length × 0.25 mm i.d. × 0.25 μm film thickness, J&W Scientific, USA) was employed for GC-MS analysis of sugars by MetWare (http://www.metware.cn/). Helium was used as carrier gas, at a flow rate of 1 mL/min. Injections were made in the split mode with a split ratio 5:1 and the intection volume was 1 μL. The oven temperature was held at 160°C for 1 min, and then raised to 200°C at 6°C/min, raised to 270°C at 10°C/min, raised to 300°C at 5°C/min, raised to 320°C at 20°C/min and held at the temperature for 5.5 min. All samples were analyzed in selective ion monitoring mode. The ion source and transfer line temperature were 230°C and 280°C, respectively (Lowell et al., 1989; Medeiros and Simoneit, 2007; Gomez-Gonzalez et al., 2010).

### Transcriptome sequencing

Total RNA was extracted from 0.5 g sample using RNAprep Pure Plant Kit (Tiengen, Beijing, China), and RNA purity was detected using a NanoPhotometer® spectrophotometer (IMPLEN, CA, USA). cDNA libraries were prepared using the NEBNext®Ultra™RNA Library Prep Kit (Illumina, San Diego, CA, USA). The NovaSeq 6000 sequencing system (Illumina) was used for sequencing with 150 bp paired-ends by Novogene Company (Beijing, China) (Modi et al., 2021). The raw data of each sample was more than 6 GB. RNA-seq data were uploaded to NCBI and can be accessed through BioProject accession number PRJNA883560.

### RNA-Seq data analysis

The Chinese chestnut reference genome and gene model annotation files were downloaded from the website (https://github.com/yongshuai-sun/hhs-omei) (Sun et al., 2020). Clean reads were obtained by filtering raw reads using Perl scripts. Then, the clean reads were compared with the reference genome using Hisat2 v2.0.5 software, and the number of reads mapped to each gene was counted by features v1.5.0-p3 in the subread software to obtain the FPKM value. The differentially expressed genes (DEGs) were analyzed by OmicShare tool (www.omicshare.com/tools ) (|log2 (fold change)|≥1, FDR ≤ 0.05). KEGG enrichment analysis was performed using STRING database (http://www.string-db.org). Gene expression data were normalized and plotted using Tbtools V1.09876 software.

### The enzyme activity of SUS

SUS was assayed in both the synthetic and cleavage directions. One gram of the frozen powder was resuspended at 4°C in 5 ml of 100 mM HEPES (pH 7.5), 2 mM EDTA and 5 mM dithiothreitol. The medium for SUS synthesis contained in a 0.5 mL volume: extract, 80 mM Hepes (pH 8.5), 5 mm KCN, 5 mm NaF, 100 mM fructose, and 15 mM UDPG. The medium for SUS cleavage in a similar volume consisted of extract, 80 mM MES (pH 5.5), 5 mM NaF, 100 mM sucrose, and 5 mM UDP. Reactions proceeded for 15 min at 30°C and were terminated by boiling for 1 min. UDP production was quantified by measurement of pyruvate kinase-specific loss of NADH in the presence of lactic dehydrogenase, and UDPG production was quantified by measuring UDPG dehydrogenase-specific synthesis of NADH (Baroja-Fernández et al., 2009).

### Co-expression network analysis

Weighted gene co-expression network analysis (WGCNA) (V1.69) in R software package was used to construct the gene co-expression network, using the signed-hybrid network type. The co-expression network was mapped using Cytoscape V3.7.1 (https://cytoscape.org/) software. The description of gene function comes from the STRING database (Szklarczyk et al., 2019).

### qRT-PCR analysis

Real-time quantitative PCR experiments were performed on ABI 7500 Real-Time PCR system (Applied Biosystems Inc., Foster City, CA, USA) with TB Green Premix Ex Taq (Takara). The instrument settings were: 95°C for 300 s; 40 PCR cycles, with each cycle set at 95°C for 10 s and 60°C for 30 s. The specific primer information was shown in **Table S1**, in which the *18S* gene of Chinese chestnut was used as the reference gene. The relative expression levels were calculated using the 2^-ΔΔCt^ method. Three biological replicates were performed.

## Results and discussion

### Morphological characteristics and sugar content

**Figure 1:**
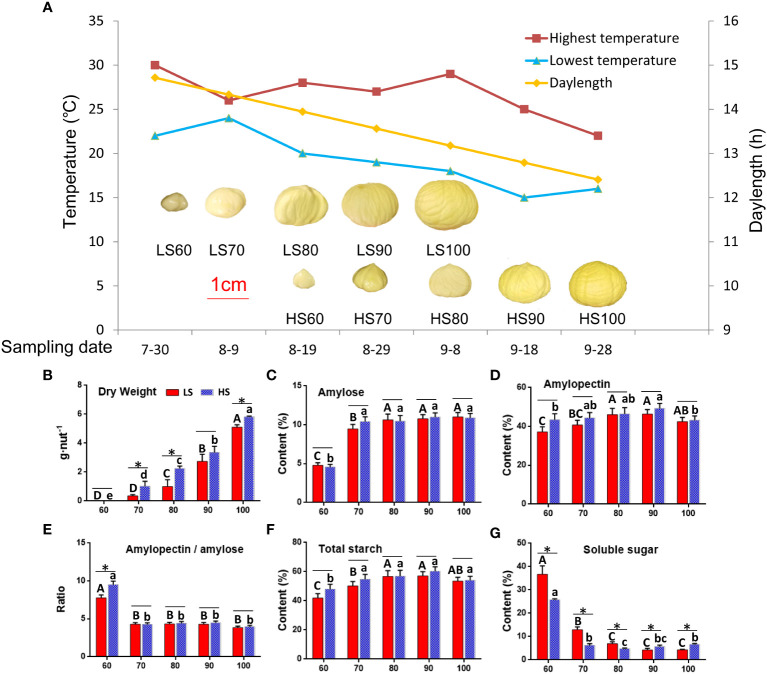
Morphological characteristics and sugar contents in two Chinese chestnut cultivars during embryo development: **(A)** morphological characteristics at 60−100 days after flowering (DAF). ‘Yanshanzaofeng’ (LS) has female flowers in full bloom on June 5, and ‘Yanlong’ (HS) has female flowers in full bloom on June 25. The ruler is 1 cm. **(B–G)** Dry weight and sugar contents in the developing embryo. Values are means ± standard deviation (SD), *n* = 3. The statistical significance between cultivars was determined by the Student’s t test (*p < 0.05). The statistical significance between stages of the same cultivar was evaluated by one-way analysis of variance (ANOVA) with Duncan’s multiple comparison test (p < 0.05), and capital letters indicate LS and Lowercase letters indicate HS. The same letters used within the same cultivar at different stages indicate no significant difference at the p≥ 0.05 level.

We have sampled five nut development stages of the two cultivars under the same site conditions. The mature stage of LS was 20 days earlier than HS, and the development temperature of HS is lower than that of LS (**Figure 1A**). The accumulation of nutrients in two cultivars was mainly carried out from 60 to 100 DAF. At 100 DAF, the dry weight of HS was 6.77g, which was 0.49g heavier than in LS (**Figure 1B**). The changes of amylose (**Figure 1C**), amylopectin (**Figure 1D**), ratio of amylopectin to amylose (**Figure 1E**) and total starch (**Figure 1F**) contents in the two cultivars were similar. It is worth noting that the soluble sugar of HS is 9%, which was 1.5 times higher than LS at the mature stage (**Figure 1G**). The soluble sugar in HS increased rapidly from 6.46% to 6.84% at 90-100 DAF, and the amylopectin decreased from 56.03% to 44.77%. The increase of soluble sugar in HS from 90 to 100 days was due to the decomposition of amylopectin. The difference of temperature and daylength during nut development may be one of the reasons for the difference in soluble sugar content in two cultivars.

### Changes in the soluble sugar composition

**Figure 2:**
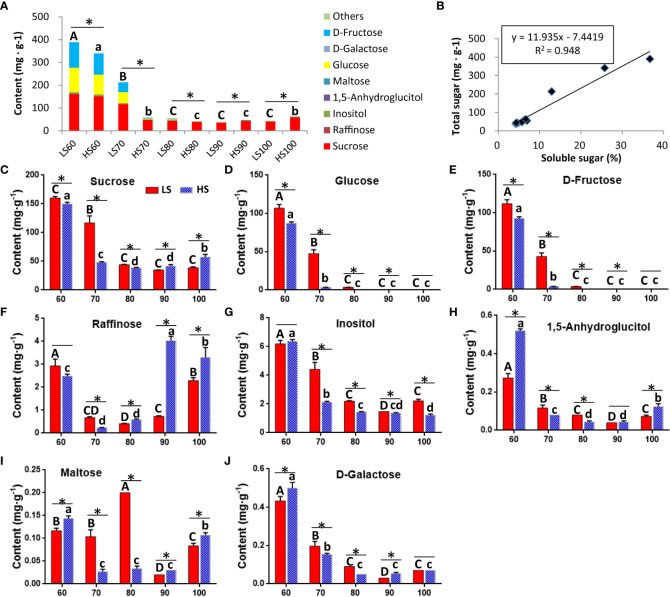
The changes of soluble sugar composition in two Chinese chestnut cultivars during embryo development: **(A)** The total content of all soluble sugar metabolites. **(B)** Correlation analysis between total content of all soluble sugar metabolites and soluble sugar content (**Figure 1G**). **(C–J)** The changes of the soluble sugar metabolites in the developing embryo. Values are means ± standard deviation (SD), *n* = 3. The statistical significance between cultivars was determined by the Student’s t test (*p < 0.05). The statistical significance between stages of the same cultivar was evaluated by ANOVA with Duncan’s multiple comparison test (p < 0.05), and capital letters indicate LS and Lowercase letters indicate HS. The same letters used within the same cultivar at different stages indicate no significant difference at the p≥ 0.05 level.

We tested a total of 32 kinds of sugar metabolites, of which 30 kinds of metabolites were detected, and the two metabolites (D-Arabinitol and L-Rhamnose) were not detected (**Table 1**). The content of the total sugar metabolites was related to soluble sugar content (**Figure 2B**), indicating that the experimental data was reliable. The results showed that the soluble sugar composition changed obviously with embryo development. The soluble sugar was mainly sucrose, glucose and fructose at 60-70 DAF (**Figure 2A**). In addition, the content of glucose (**Figure 2D**) and fructose (**Figure 2E**) was high (> 87.01 mg/g) at 60 DAF but low (<3.68 mg/g) at 80-100 DAF. In mature chestnuts, the soluble sugar was mainly sucrose (> 38.67 mg/g), and the content of other sugar metabolites was very low (<3.30 mg/g). At 90-100 DAF, the sucrose content in HS was 1.5 times higher than that in LS (**Figure 2C**). In addition, the content of raffinose (**Figure 2F**), 1,5-Anhydroglucitol (**Figure 2H**) and maltose (**Figure 2I**) was higher in HS than that in LS at mature stage. The content of inositol (**Figure 2G**) was higher in LS than that in HS at mature stage. During ripening, the increased content of these soluble sugar metabolites will make the chestnuts sweeter.

**Table 1:** Identification of sugar-related metabolites in the developing Chinese chestnut embryo.

Compounds	LS60	LS70	LS80	LS90	LS100	HS60	HS70	HS80	HS90	HS100
**Sucrose**	**160.41**	**116.97**	**44.06**	**34.92**	**38.67**	**149.94**	**47.94**	**38.53**	**41.57**	**57.44**
**D-Fructose**	**111.78**	**42.96**	3.68	0.24	0.07	**92.69**	3.68	0.43	0.07	0.06
**Glucose**	**106.99**	**47.53**	3.52	0.20	0.08	**87.01**	3.40	0.28	0.08	0.08
**Inositol**	6.19	4.41	2.20	1.47	2.22	6.36	2.12	1.44	1.35	1.22
**Raffinose**	2.93	0.67	0.42	0.73	2.29	2.48	0.23	0.58	4.03	3.30
**D-Galactose**	0.44	0.20	0.09	0.03	0.07	0.50	0.15	0.05	0.05	0.07
**1,5-Anhydroglucitol**	0.27	0.12	0.08	0.04	0.07	0.52	0.08	0.05	0.04	0.12
**Maltose**	0.11	0.10	0.20	0.02	0.08	0.14	0.03	0.03	0.03	0.11
**D-Mannose**	0.17	0.09	0.05	0.02	0.02	0.19	0.07	0.03	0.02	0.02
**D-Mannose-6-phosphate sodium salt**	0.05	0.03	0.03	0.03	0.03	0.05	0.03	0.03	0.03	0.03
**D-Xylulose**	0.09	0.05	0.01	0.01	0.01	0.11	0.01	0.01	0.01	0.01
**Methyl beta-D-galactopyranoside**	0.05	0.03	0.02	0.01	0.02	0.11	0.02	0.01	0.01	0.03
**Barium D-ribose-5-phosphate**	0.03	0.02	0.02	0.02	0.02	0.03	0.02	0.02	0.02	0.02
**Cellobiose**	0.06	0.04	0.02	0.01	0.01	0.06	0.01	0.01	0.01	0.01
**D-Sorbitol**	0.03	0.06	0.02	0.00	0.00	0.09	0.01	0.00	0.00	0.00
**2-Acetamido-2-deoxy-D-glucopyranose**	0.03	0.02	0.02	0.02	0.01	0.04	0.02	0.02	0.02	0.02
**D-Xylose**	0.04	0.03	0.02	0.00	0.00	0.07	0.02	0.01	0.00	0.00
**Lactose**	0.03	0.02	0.01	0.01	0.02	0.02	0.00	0.00	0.00	0.00
**L-Fucose**	0.02	0.01	0.01	0.01	0.01	0.03	0.01	0.01	0.01	0.01
**D-Arabinose**	0.03	0.01	0.01	0.00	0.00	0.04	0.01	0.00	0.00	0.00
**Levoglucosan**	0.01	0.01	0.01	0.01	0.01	0.01	0.01	0.01	0.01	0.01
**Trehalose**	0.03	0.01	0.00	0.00	0.00	0.02	0.00	0.00	0.00	0.00
**D-Glucuronic acid**	0.01	0.01	0.01	0.00	0.00	0.02	0.01	0.00	0.00	0.00
**Deoxyglucose**	0.01	0.01	0.00	0.00	0.01	0.01	0.00	0.01	0.01	0.01
**D-Ribose**	0.02	0.00	0.00	0.00	0.00	0.02	0.00	0.01	0.00	0.00
**D-Ribono-1,4-lactone**	0.01	0.01	0.00	0.00	0.00	0.02	0.00	0.00	0.00	0.01
**Xylitol**	0.00	0.01	0.00	0.01	0.01	0.00	0.00	0.01	0.01	0.01
**Phenylglucoside**	0.00	0.00	0.00	0.00	0.00	0.00	0.00	0.00	0.00	0.00
**D-Galacturonic acid**	0.01	0.00	0.00	0.00	0.00	0.01	0.00	0.00	0.00	0.00
**2-Deoxy-D-ribose**	0.00	0.00	0.00	0.00	0.00	0.00	0.00	0.00	0.00	0.00
**D-Arabinitol**	–	–	–	–	–	–	–	–	–	–
**L-Rhamnose**	–	–	–	–	–	–	–	–	–	–

The metabolites with values > 100 are highlighted with bold font. A total of 32 kinds of sugar metabolites were tested, and two of them were not detected.

### Transcriptome sequencing and clustering of DEGs

Thirty libraries from Chinese chestnut embryos were sequenced. We obtained a total of 1.41 billion base pairs, with an average of 47,036,059 raw reads and 45,312,612 clean reads per sample. The average ratio of clean reads to raw reads was 96.33% (**Table S2**). The clean reads are made freely available in the NCBI (accession number: PRJNA883560).

**Figure 3:**
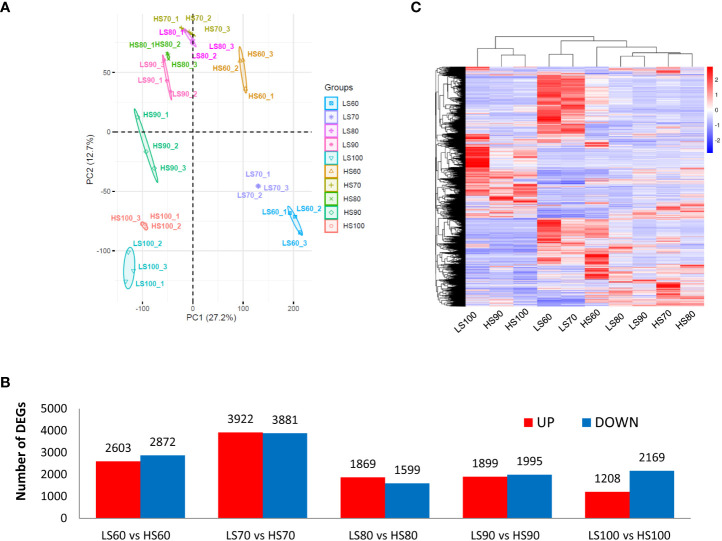
PCA of samples and cluster analysis of DEGs: **(A)** PCA of all 30 samples was conducted based on RNA-seq FPKM. **(B)** Pairwise comparison between two cultivars at same stage. We identified a total of 14,430 DEGs in **Table S3**. **(C)** Cluster analysis of DEGs based on mean FPKM. The color scale (−3 to 3) represents the calculated Z-score.

A total of 42,740 unigenes were identified from transcriptome data. Principal components analysis (PCA) of all 30 samples was conducted based on RNAseq FPKM, and two principal components were found to explain 39.9% of the overall variance (27.2% and 12.7% for PC1 and PC2, respectively). The three samples at the same stage were close to each other, indicating that there was a high consistency between the three biological replicates (**Figure 3A**). A total of 14,430 DEGs were identified by pair comparison between two cultivars at each stage (**Figure 3B**). The cluster analysis of DEGs was shown in **Figure 3C**. The results showed that the 30 samples could be divided into three groups: LS60 and HS60 constituted the first group, LS70, LS80, LS90, HS70 and HS80 constituted the second group, and LS100, HS90 and HS100 formed the third group.

### Trend analysis of DEGs in embryo

**Figure 4:**
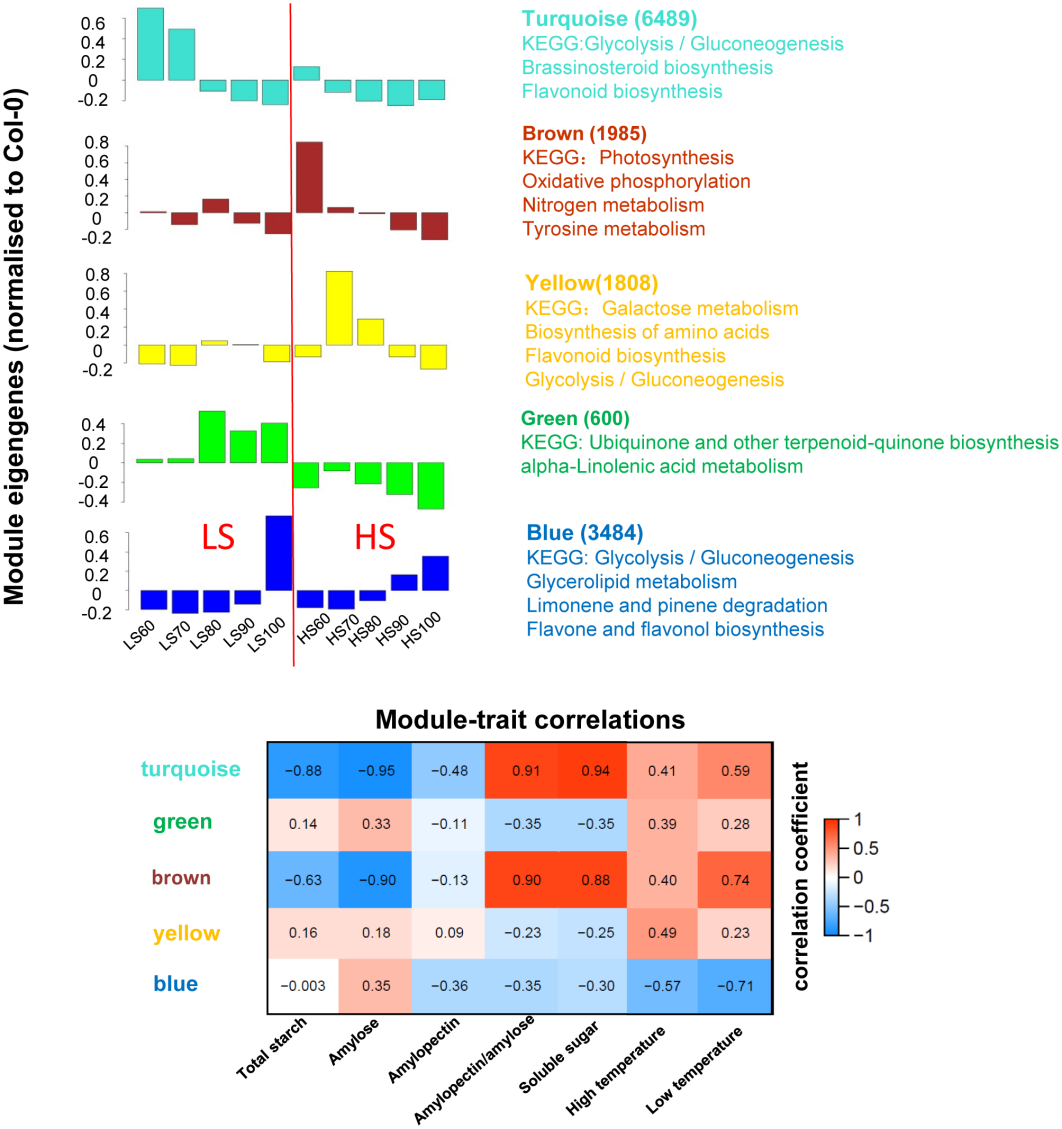
WGCNA of RNA-seq and traits: **(A)** WGCNA was calculated by 30 samples and the 14,430 DEGs were classified into 5 modules. Columns represent module eigengene of mean values. The number of DEGs and KEGG pathways for each module were listed; **(B)** expression patterns of the modules were correlated to physiological data. The numbers in each colored box give the values for correlation coefficient.

In order to further understand the mechanism of sugar synthesis in Chinese chestnut embryos, we focused on the expression trend of DEGs. The 14,430 DEGs were divided into five modules using WGCNA (**Figure 4A**), and the number of DEGs and KEGG pathways of each module were listed in each module. The largest module (turquoise) contained 6489 DEGs whose expression was highest in the LS60 sample, which included genes related to glycolysis/gluconeogenesis, brassinosteroid biosynthesis, and flavonoid biosynthesis. The second largest module (blue) contained 3484 DEGs whose expression was the highest expression in the LS100 sample, which included genes related to glycolysis/gluconeogenesis, glycerolipid metabolism, limonene and pinene degradation, and flavone and flavonol biosynthesis. The third largest module (brown) contained 1985 DEGs whose expression was the highest expression in the HS60 sample, which included genes related to photosynthesis, oxidative phosphorylation, nitrogen metabolism, tyrosine metabolism.

In order to understand the relationship between the gene expression patterns and metabolites, association analysis was performed using WGCNA (**Figure 4B**). Sugar content was highly correlated with turquoise and brown modules. Combined with KEGG pathway and module-trait correlation analysis, turquoise, blue and brown modules were associated with sugar biosynthesis.

### Identification of genes related to sucrose degradation and starch synthesis

The sucrose produced by photosynthesis will be stored in the chestnut as starch. As shown in **Table S3,** a total of 66 unigenes related to starch synthesis were identified, of which 15 belong to the turquoise module, 9 belong to the blue module, and 4 belongs to the other module. Most of the genes related to starch synthesis were highly expressed at 70 DAF. Some genes such as *SUS*, *GPT* and *GBSS* had FPKM values >1000 in our transcriptome data. These highly expressed genes may play an important role in starch synthesis in Chinese chestnut embryos. The variation trend of these genes related to starch synthesis was similar to that reported in previous articles (Zhang et al., 2015; Shi et al., 2021), indicating that the regulation of these genes in chestnut cultivars was similar.

**Figure 5:**
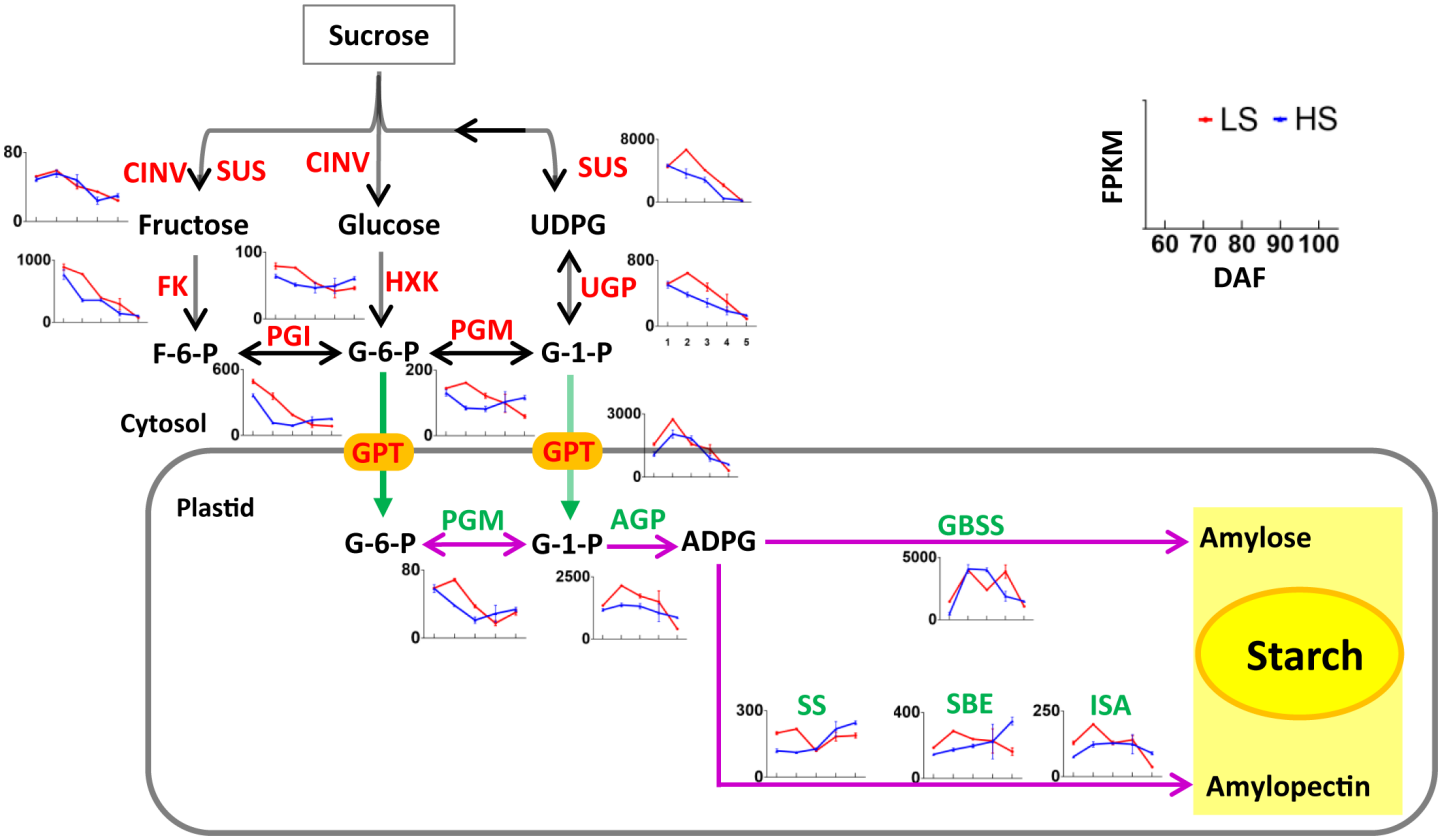
Nonpartitioned expression pattern of genes related to sucrose degradation and starch synthesis. Symbols represent mean FPKM values and the horizontal axis represents 60, 70, 80, 90, and 100 DAF. Red line represents LS, and blue line represents HS (high sugar cultivar). The two-way arrow indicates a reversible reaction, and the one-way arrow indicates an irreversible reaction. The information of genes are listed in **Table S3**.

**Figure 6:**
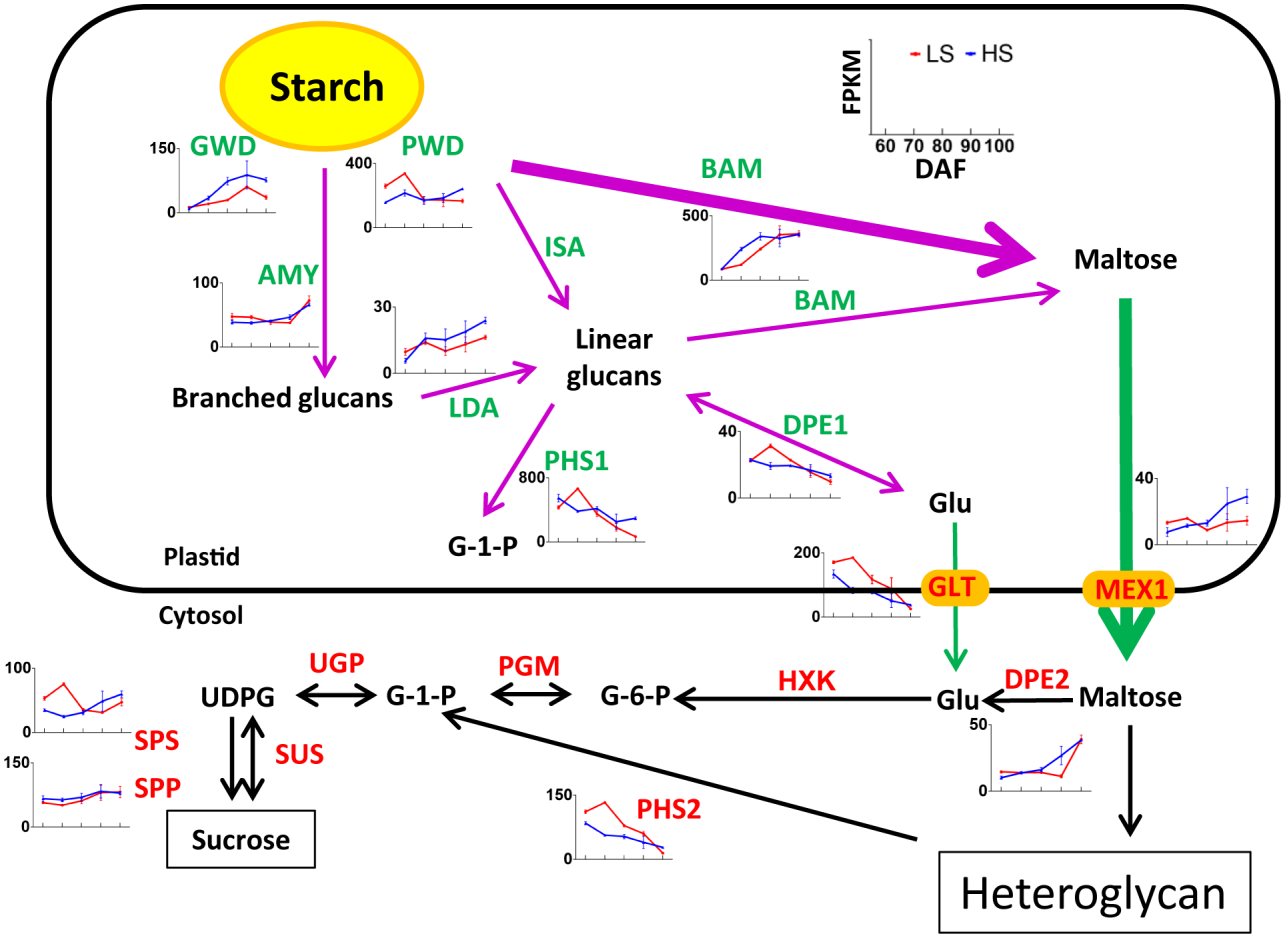
Nonpartitioned expression pattern of genes related to sucrose degradation and starch synthesis. Symbols represent mean FPKM values and the horizontal axis represents 60, 70, 80, 90, and 100 DAF. Red line represents LS, and blue line represents HS (high sugar cultivar). The two-way arrow indicates a reversible reaction, and the one-way arrow indicates an irreversible reaction. The information of genes are listed in **Table S4**.

At 70-80 DAF, the expression level of genes related to starch synthesis (i.e., *SUS*, *PGI*, *UGP*, *PGM* and *AGP*) was higher in LS than that in HS (**Figure 5**). At 100 DAF, the expression level of genes related to starch synthesis (i.e., *HXK*, *PGI*, *PGM*, *AGP*, *SS*, *SBE*, *ISA*) was higher in HS than that in LS (**Figure 6**). This indicates that the sugar metabolism of HS was more active than that of LS at mature stage.

### Identification of genes related to starch degradation and sucrose synthesis

Starch degradation during ripening is a key additional process for sugar accumulation and sweetness in fruit (Liu et al., 2021). As shown in **Table S4**, a total of 33 unigenes related to starch degradation were identified, of which 6 belong to the turquoise module, 6 belong to the blue module, and 3 belongs to the other module. Some genes such as *PWD*, *BAM*, *PHS1*, *GLT*, *PHS2* had FPKM values >100 in our transcriptome data. These highly expressed genes may play an important role in starch degradation in Chinese chestnut embryos. At 100 DAF, the expression level of genes related to starch degradation (i.e., *GWD*, PWD, *LDA*, *ISA*, *PHS1*, *MEX1*) was higher in HS than that in LS (**Figure 6**). This indicated that starch degradation of HS was more intense than that of LS at mature stage, which was consistent with soluble sugar content.

It is worth noting, the expression level of *GWD* genes was higher in HS than that in LS at 70-100 DAF. GWD catalyzes the phosphorylation of amylopectin to form phosphoglucan, which is a key enzyme in the degradation of plant starch (You et al., 2020). In rice research, GWD can improve the yield and quality characteristics, which has great application potential (Wang et al., 2021). Therefore, GWD may play an important role in starch degradation of chestnut at mature stage.

### Analysis of *SUS* gene

**Figure 7:**
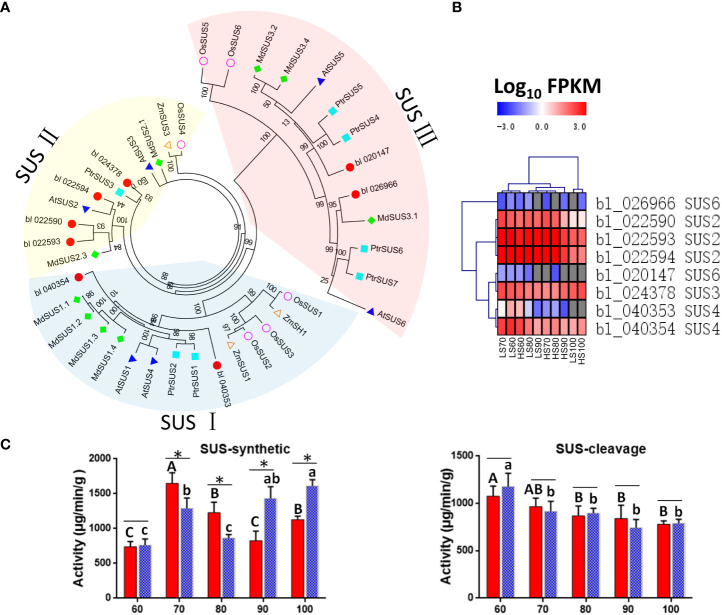
Putative sucrose synthase (SUS) unigenes identified in Chinese chestnut: **(A)** phylogenetic analysis of CmSUSs (*Castanea mollissima*) and AtSUSs (Arabidopsis thaliana) with Protein sequences. The Protein sequences were aligned using ClustalX2, and the neighbor-joining (NJ) tree was constructed with the program MEGA 6.0. A total of 1000 bootstrap replications were carried out to indicate reliability. **(B)** Heat map showing expression levels of *CmSUSs*. **(C)** Enzyme activity of SUS in two Chinese chestnut cultivars during embryo development. Values are means ± standard deviation (SD), n = 3. The statistical significance between cultivars was determined by the Student’s t test (*p < 0.05). The statistical significance between stages of the same cultivar was evaluated by ANOVA with Duncan’s multiple comparison test (p < 0.05), and capital letters indicate LS and Lowercase letters indicate HS. The same letters used within the same cultivar at different stages indicate no significant difference at the p≥ 0.05 level.

Sucrose synthase (SUS) is the key enzyme of sucrose metabolism, which has the function of decomposition and synthetic sucrose (Stein and Granot, 2019). In this study, we identified a total of 8 *SUSs* in Chinese chestnut, including three *SUS2*, one *SUS3*, two *SUS4*, and two *SUS6* genes. Among these 8 *SUSs*, 2, 4, and 2 genes belong to the SUSI subfamily, SUSII subfamily, SUSIII subfamily, respectively (**Figure 7A**). The *SUS2* (bl_022590, bl_022593 and bl_022594), *SUS3* (bl_024378), and *SUS4* (bl_040354) genes were highly expressed (FPKM> 100) (**Table S3**), which may play an important role in sucrose content during Chinese chestnut embryo development.

The *SUS6* gene were lowly expressed, but the *SUS2* were highly expressed in developing Chinese chestnut embryos. The expression heat map revealed that three *SUS2* genes (bl_022590, bl_022593 and bl_022594) could be grouped into a category, with the high expression levels observed at 60-90 DAF (**Figure 7B**). It is noteworthy that the expression level of *SUS3* (bl_024378) was higher in HS than that in LS at 90-100 DAF.

In addition, we measure the enzyme activity of SUS. The enzyme activity of SUS-cleavage continues to decrease during embryo development, and there is no significant difference between the two cultivars (**Figure 7C**). However, the enzyme activity of SUS-synthetic was significantly different between the two cultivars. The enzyme activity of SUS-synthetic was higher in LS than that in HS at 70-80 DAF, while was higher in HS than that in LS at 90-100 DAF. The enzyme activity of SUS changes were consistent with changes of sucrose content in the two cultivars.

One study showed that after 48 h cold stress treatment of *Arabidopsis thaliana* (Baslam et al., 2017), the expression levels of *AtSus2*, *AtSus4*, *AtSus5* and *AtSus6* were not affected, while the expression levels of *AtSus1* and *AtSus3* were 24.5 times and 5.5 times of those in the untreated group, respectively. The changes of these genes were consistent with our results, and cold stress may affect *SUS3* (bl_024378) gene expression, and then change the enzyme activity of SUS-synthetic in Chinese chestnut.

### ABA promote the increase of soluble sugar

**Figure 8:**
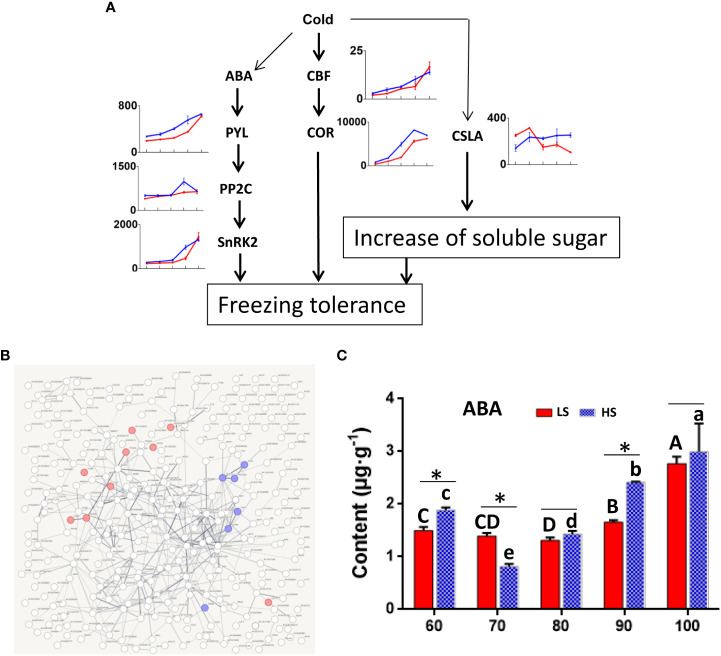
Effect of low temperature on soluble sugar in Chinese chestnut. **(A)** Identification of genes related to cold stress. Red line represents LS, and blue line represents HS (high sugar cultivar). The information of genes are listed in **Table S5**. **(B)** Co-expression network for *COR* (bl_030433). The information of genes are listed in **Table S6**. Red genes were associated with ABA signaling pathway and blue genes were associated with peroxisome. **(C)** ABA content in two Chinese chestnut cultivars during embryo development. ABA content was carried out by ELISA (Liu et al., 2008). Values are means ± standard deviation (SD), n = 3. The statistical significance between cultivars was determined by the Student’s t test (*p < 0.05). The statistical significance between stages of the same cultivar was evaluated by ANOVA with Duncan’s multiple comparison test (p < 0.05), and capital letters indicate LS and Lowercase letters indicate HS. The same letters used within the same cultivar at different stages indicate no significant difference at the p≥ 0.05 level.

Some studies have shown that cold stress can promote the increase of soluble sugar in plants (Zhang et al., 2011; Ding et al., 2019) (**Figure 8A**). In addition, COR is a key gene in response to cold stress, which encoded hydrophilic peptide can protect the cells from freezing damage. Our data showed that the expression level of COR gene was higher in HS than that in LS at 60-100 DAF. Notably, *COR* (bl_030433) and *GWD* (bl_017818) were co-expressed, which may reflect the correlation between starch degradation and low temperature. Then, we analyzed the 412 genes co-expressed with *COR* (bl_030433) by string analysis (**Figure 8B**). There were 9 genes associated with ABA signaling pathway and 8 genes associated with peroxisome. ABA and peroxide may play an important role in starch decomposition during Chinese chestnut ripening.

Therefore, we also analyzed ABA content in two Chinese chestnut cultivars during embryo development (**Figure 8C**). At 90 DAF, ABA content in HS was higher than that in LS. Correspondingly, the expression level of genes related to ABA (i.e., *PYL*, *PP2C* and *SnRK2*) was also higher in HS than in LS (**Figure 8A**). In addition, the expression of *CSLA* gene related to polysaccharide synthesis was higher in HS than in LS at 80-100 DAF. Some studies have also shown that ABA plays an important role in promoting starch degradation and sucrose synthesis in fruits. Exogenous ABA treatment could promote mango ripening and increase soluble sugar (Wu et al., 2022).

### qPCR analysis of sugar-related genes

**Figure 9:**
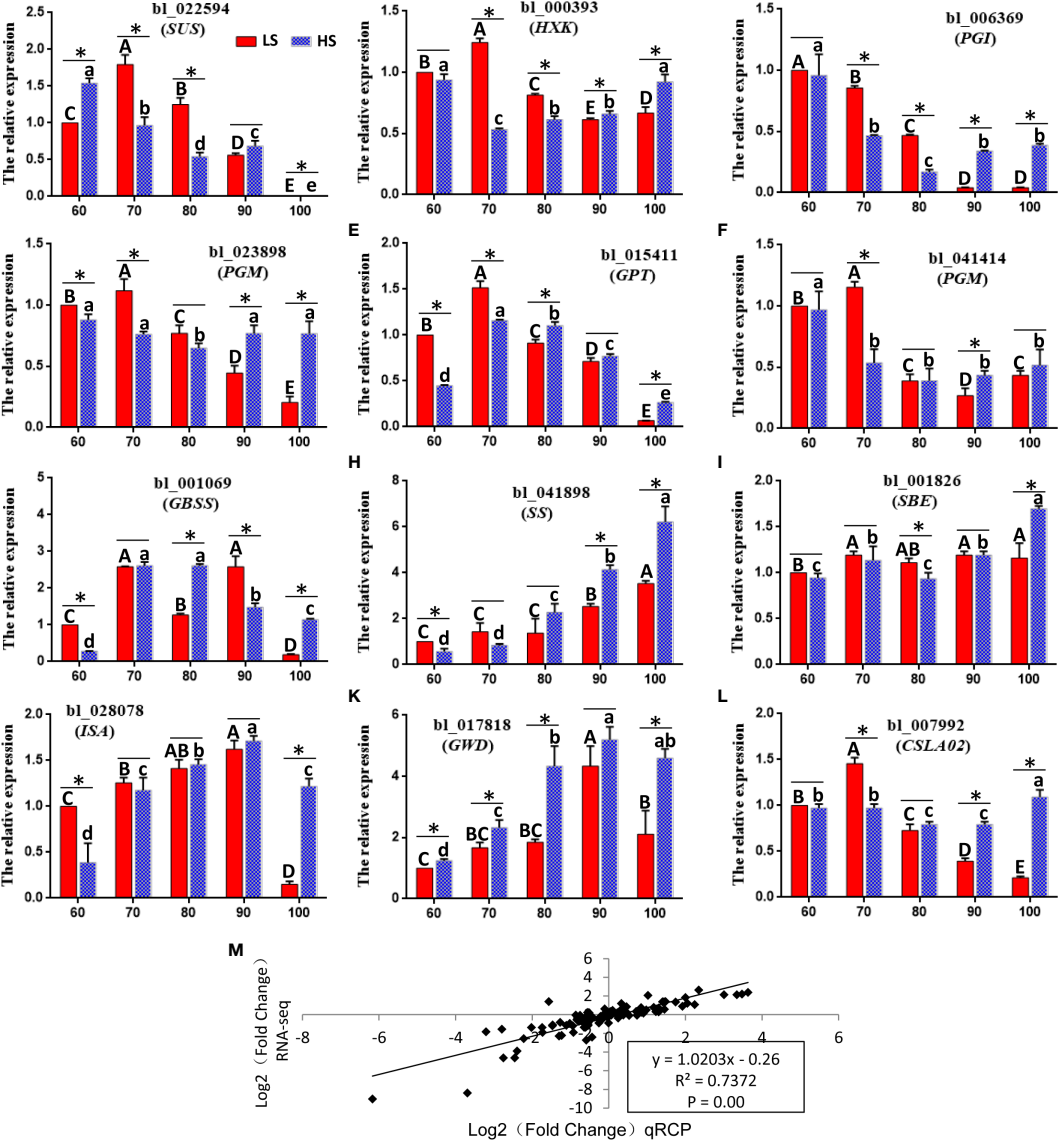
qPCR validation of differential expression. **(A–L)** qPCR of 12 important genes associated with sugar biosynthesis. Symbols represent mean values and short vertical lines indicate SE (*n* = 3). Same letters stand for statistically insignificance (p > 0.05). The unit of measure on the x-axis is sample. **(M)** A comparison of the gene expression ratios obtained from RNA-seq data and q-PCR. The statistical significance between cultivars was determined by the Student’s t test (*p < 0.05). The statistical significance between treatments was evaluated by ANOVA with Duncan’s multiple comparison test (p < 0.05). Capital letters indicate LS. Lowercase letters indicate HS. The same letters used within the same cultivar at different stages indicate no significant difference at the p≥ 0.05 level.

In order to verify the relative expression pattern of unigenes in RNA-seq analysis, twelve key genes related to sugar metabolism were analyzed by qPCR (Panels A-L) (**Figure 9**). Panel M shows that RNA-seq is highly correlated with qPCR data (R^2^ = 0.74, p <0.01), indicating that the expression data obtained by RNA-seq is reliable. Among all 12 genes, the expression level of 10 genes (*HXK*, *PGI*, *PGM*, *GPT*, *GBSS*, *SS*, *SBE*, *ISA*, *GWD* and *CLSC20*) was higher in HS than that in LS at 100 DAF, indicating that the active sugar metabolism was related to the increase in the content of soluble sugar in LS at the mature stage.

## Conclusions

**Figure 10:**
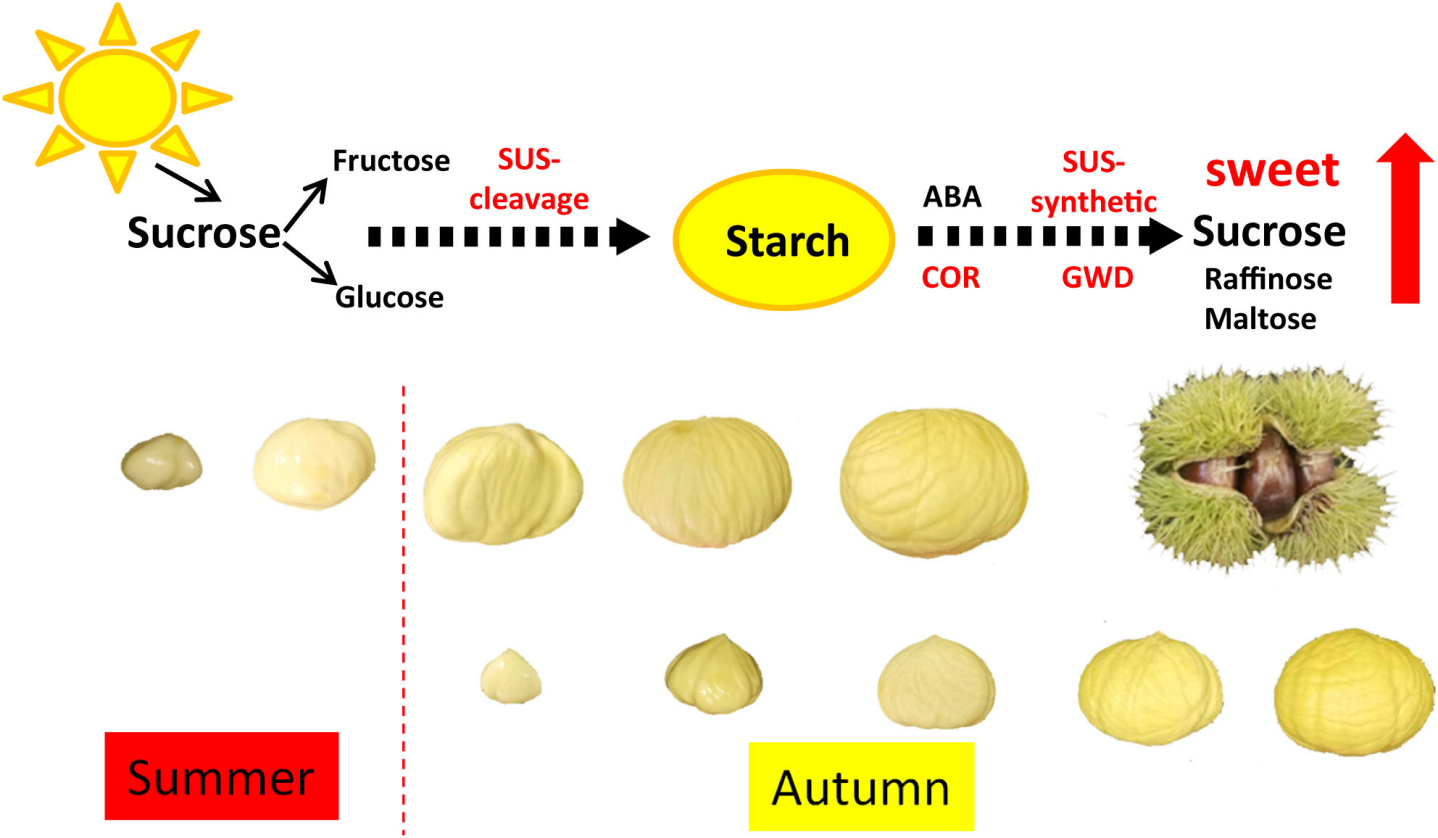
Regulation model of sucrose and starch conversion in Chinese chestnut. During Chinese chestnut development, sucrose is synthesized by photosynthesis and transported to the embryo, and sucrose is decomposed into fructose and glucose to synthesize starch. At the mature stage, ABA content in chestnut was increased. ABA further stimulated the expression of genes *(*i.e., *SUS* and *GWD)*, and then promoted the decomposition of starch into sucrose, which increased the sweetness of chestnut kernels.

In this study, we investigated the molecular mechanism of the difference in sugar accumulation between two Chinese chestnut cultivars. Metabolome and transcriptome analysis showed that starch degradation was an important pathway for the increase of soluble sugar content in chestnut at mature stage. Furthermore, the enzyme activity of SUS-synthetic and ABA content were higher in HS than those in LS at 90 DAF. Based on our findings, we preliminarily established the regulation model of sucrose and starch conversion in chestnut (**Figure 10**). During chestnut development, sucrose is synthesized by photosynthesis and transported to the embryo, and sucrose is decomposed into fructose and glucose to synthesize starch. At the mature stage, ABA content in chestnut was increased. ABA further stimulated the expression of genes *(*i.e., *SUS* and *GWD)*, and then promoted the decomposition of starch into sucrose, which increased the sweetness of chestnut kernels. Our study analyzed the composition and molecular synthesis mechanism of sugar in Chinese chestnut embryos, and provided a new insight into the regulation pattern of high sugar accumulation in Chinese chestnut nuts. It is noteworthy that HS has typical late ripening behavior, while LS has late ripening behavior in this study. Whether mature behavior and low temperature are related to the sugar content of chestnuts deserve further research.

## Data availability statement

The datasets presented in this study can be found in online repositories. The names of the repository/repositories and accession number(s) can be found in the article/**Supplementary Material.**

## Author contributions

RH and YY designed the experiments. RH, FP, DW, FC, and CG carried out the experiments. LY, JZ, and YY analyzed the data. RH and FP drafted the manuscript. All authors contributed to the article and approved the submitted version.
